# Transglutaminase-2 facilitates extracellular vesicle-mediated establishment of the metastatic niche

**DOI:** 10.1038/s41389-020-0204-5

**Published:** 2020-02-13

**Authors:** Aparna Shinde, Juan Sebastian Paez, Sarah Libring, Kelsey Hopkins, Luis Solorio, Michael K. Wendt

**Affiliations:** 1 Department of Medicinal Chemistry and Molecular Pharmacology, Purdue University, West Lafayette, IN 47907 USA; 2 Department of Biomedical Engineering, Purdue University, West Lafayette, IN 47907 USA; 3 Purdue Center for Cancer Research, Purdue University, West Lafayette, IN 47907 USA

**Keywords:** Breast cancer, Membrane trafficking

## Abstract

The ability of breast cancer cells to interconvert between epithelial and mesenchymal states contributes to their metastatic potential. As opposed to cell autonomous effects, the impact of epithelial–mesenchymal plasticity (EMP) on primary and metastatic tumor microenvironments remains poorly characterized. Herein we utilize global gene expression analyses to characterize a metastatic model of EMP as compared to their non-metastatic counterparts. Using this approach, we demonstrate that upregulation of the extracellular matrix crosslinking enzyme tissue transglutaminase-2 (TG2) is part of a novel gene signature that only emerges in metastatic cells that have undergone induction and reversion of epithelial–mesenchymal transition (EMT). Consistent with our model system, patient survival is diminished when primary tumors demonstrate enhanced levels of TG2 in conjunction with its substrate, fibronectin. Targeted depletion of TG2 inhibits metastasis, while overexpression of TG2 is sufficient to enhance this process. In addition to being present within cells, we demonstrate a robust increase in the amount of TG2 and crosslinked fibronectin present within extracellular vesicle (EV) fractions derived from metastatic breast cancer cells. Confocal microscopy of these EVs suggests that FN undergoes fibrillogenesis on their surface via a TG2 and Tensin1-dependent process. Upon in vivo administration, the ability of tumor-derived EVs to induce metastatic niche formation and enhance subsequent pulmonary tumor growth requires the presence and activity of TG2. Finally, we develop a novel 3D model of the metastatic niche to demonstrate that conditioning of pulmonary fibroblasts via pretreatment with tumor-derived EVs promotes subsequent growth of breast cancer cells in a TG2-dependent fashion. Overall, our studies illustrate a novel mechanism through which EMP contributes to metastatic niche development and distant metastasis via tumor-derived EVs containing aberrant levels of TG2 and fibrillar FN.

## Introduction

Metastatic progression is the major driver of lethality in breast cancer^1^. Overt manifestation of macroscopic metastases is the culminating event in the multistep process of disease progression. However, recent efforts from our laboratory and others clearly indicate that molecular events take place very early in disease progression that can influence the success and failure of disseminated cells to proliferate in secondary tissues and establish metastatic disease^2^. Among these, the molecular and phenotypic aspects of epithelial–mesenchymal transition (EMT) play a key role in maximizing the metastatic potential of mammary tumors through several mechanisms^3^. Clearly, the ability of tumor cells to transition to a mesenchymal state contributes to cell invasion, drug resistance, and cell survival in response to the stresses of the primary tumor environment and upon systemic dissemination^4–6^. Following dissemination and adaptation to the new microenvironment, return to an epithelial state is consistent with an enhanced ability of cells to overcome dormancy and undergo metastatic outgrowth^3,7^. In addition to these tumor cell autonomous effects of epithelial–mesenchymal plasticity (EMP) that take place at various steps in the metastatic process, differential EMP conversion rates within the primary tumor contribute to dynamic paracrine relationships between tumor cell populations of varying epithelial or mesenchymal status. We recently termed this concept, epithelial–mesenchymal heterogeneity (EMH)^8,9^. An understudied concept of EMP and EMH includes characterization of the epithelial phenotype that remerges after carcinoma cells transition to and from a mesenchymal state^10^. Herein we sought to address the hypothesis that, following induction of EMT, tumor cells will return to an epithelial state that is similar but critically unique from their original epithelial phenotype.

A key aspect of secondary tumor formation is the ability of disseminated cells to alter their surrounding extracellular matrix (ECM) to create a niche that is capable of supporting tumor initiation within the context of a normal organ^11^. Tissue transglutaminase 2 (TG2) is a crosslinking enzyme, which similar to other transglutaminases catalyzes protein crosslinking via formation of isopeptide bonds between the epsilon-amino group of a lysine and the gamma-carboxamide group of a glutamine^12^. The ability of TG2 to crosslink various ECM proteins including laminin, collagen, and fibronectin (FN) is strongly linked to fibrosis and cancer^13,14^. In addition to functioning as freely secreted molecules, TG2 and FN have also been detected on the surface of extracellular vesicles (EVs)^15,16^. The presence of matrix proteins on the surface of EVs contributes to the organotrophic delivery of their various molecular cargoes such as proteins and RNAs belonging to their cells of origin^16,17^. Therefore, the differential makeup of EVs shed by cancer cells contributes to their ability to reprogram distant stromal cells and directly create a metastatic niche.

Herein we demonstrate that TG2 and crosslinked FN are upregulated on EVs isolated from metastatic breast cancer cells that have undergone EMT–MET (mesenchymal–epithelial transition). Furthermore, we utilize in vivo approaches and a novel three-dimensional (3D) culture model of the metastatic niche to establish that the ability of EVs to reprogram pulmonary fibroblasts to support the growth of breast cancer cells is strongly dependent on the presence and function of TG2. These results provide rationale for development of TG2-targeted biomarkers and therapeutics for the diagnosis and treatment of metastatic breast cancer.

## Results

### Global characterization of gene expression following EMP

**Fig. 1:**
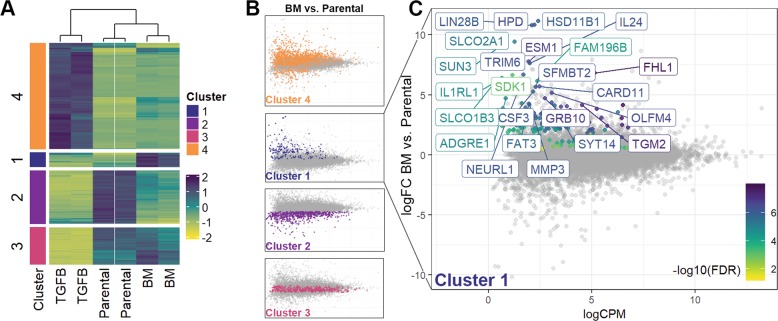
Global characterization of gene expression following epithelial–mesenchymal plasticity. **a** Dendrogram showing analysis of duplicate RNA sequencing analyses conducted on HME2 cells left untreated (Parental), treated with TGF-β1 for 4 weeks (TGFB) to induce a mesenchymal state, and TGF-β1-treated HME2 cells subcultured from a bone metastasis that formed subsequent to mammary fat pad engraftment (BM). As described in the “Materials and methods” section, gene expression changes were divided into four clusters based on differential expression between the three groups. **b** Cluster 1 was defined as genes whose expression did not change during TGF-β1-induced EMT but were significantly upregulated in the HME2-BM cells as compared to the HME2-parental cells. **c** Identification of an EMP signature of genes whose expression was significantly increased only after induction and metastatic reversion of EMT.

**Fig. 2:**
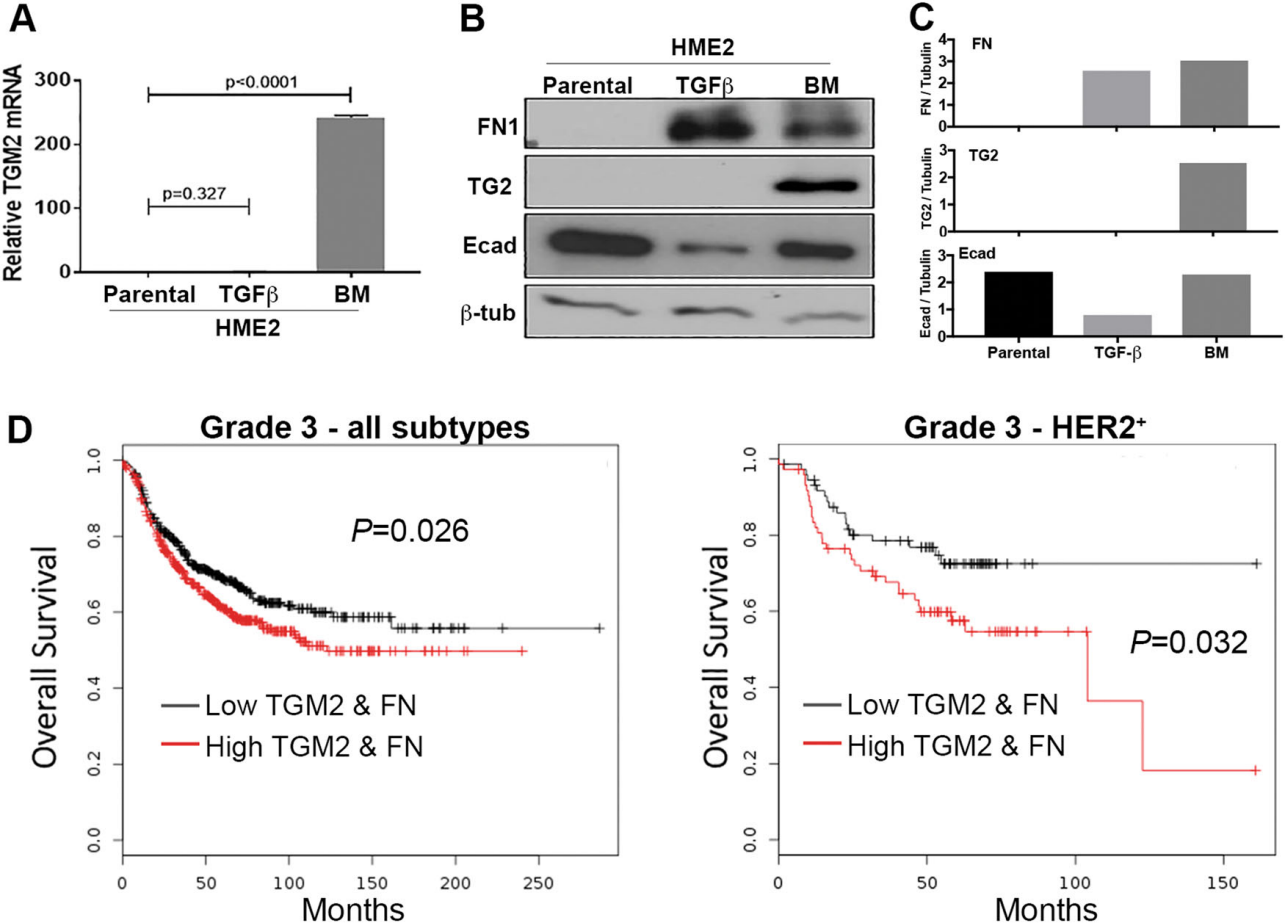
Transglutaminase-2 expression is associated with decreased patient survival. **a** Transcript levels for *TGM2* in HME2 parental, TGF-β1 treated (TGFB), and bone metastases (BM) were quantified using qRT-PCR. Data are expressed relative to HME2-parental cells and are the mean ± SE of three independent experiments resulting in the indicated *p* values. **b** Immunoblot analyses for TG2, FN1, and E-cadherin (Ecad) in HME2 parental, TGF-β1 treated (TGFB), and bone metastases (BM). Expression of β-tubulin served as a loading control. Data are representative of at least three independent experiments. **c** Densitometric analyses of the immunoblots described in **b**. **d** Comparison of overall survival between patients bearing grade 3 tumors expressing levels of TG2 and FN above (high) or below (low) the mean of the entire patient cohort. Survival curves were analyzed via a log-rank test resulting in the indicated *p* values.

Our recent studies demonstrate that induction of EMP via a 4-week treatment with transforming growth factor (TGF)-β1 followed by a 2-week withdrawal is sufficient to induce metastasis of HER2-transformed mammary epithelial cells (HME2) upon mammary fat pad engraftment^3^. Subculture of these bone metastases (HME2-BM) resulted in an epithelial cell population that is morphologically indistinguishable from the parental HME2 cells^3^. To characterize these two epithelial populations, we performed RNA sequencing analyses on the parental HME2 cells, the purely mesenchymal population that resulted immediately following TGF-β1 treatment (HME2-TGF-β), and the HME2-BM cells (GSE115255). Analysis of these gene expression data clearly indicated that long-term TGF-β1 treatment induced a gene expression profile that is characteristic of EMT and very unique from the related epithelial states of the HME2 parental and HME2-BM populations (Fig. 1a). Further analysis revealed a set of genes whose expression does not change during the onset of the mesenchymal phenotype induced by TGF-β1 but only becomes significantly upregulated following reversion to the secondary epithelial state characteristic of the HME2-BM cells (Cluster #1; Fig. 1a, b). Expression of several genes known to be involved in cancer progression were identified by this method, including HMGA2, FHL1, SLC2A3, ADAMTS1, UPP1, and TGM2. Use of quantitative real-time reverse transcription PCR (qRT-PCR) and immunoblot analyses to confirm our RNA sequencing data demonstrated increased transglutaminase-2 mRNA (TGM2) and protein (TG2) levels in HME2-BM cells as compared to the HME2-parental and TGF-β1-treated populations (Fig. 2a–c). In contrast to this mode of TG2 regulation following completion of EMP, the traditional EMT-associated gene, E-cadherin, and FN showed patterns of downregulation and upregulation during TGF-β1-induced EMT, respectively (Fig. 2b, c). Following EMP, expression of E-cadherin returned to similar levels as the parental HME2 cells, but FN levels remained elevated (Fig. 2b, c). Consistent with these findings, analysis of differential TG2 expression alone could not distinguish patient survival times, but since TG2 is known to crosslink FN in the ECM we analyzed breast cancer patient survival times based on the differential expression of both FN and TGM2^18^. Patients expressing high levels of both FN and TGM2 demonstrate decreased survival compared to those patients expressing low levels of these two genes (Fig. 2d)^9,19^. These data suggest that TG2 in conjunction with FN are clinically relevant markers of EMP whose enhanced expression within the primary tumor is consistent with metastatic disease progression.

### Transglutaminase-2 promotes breast cancer metastasis

**Fig. 3:**
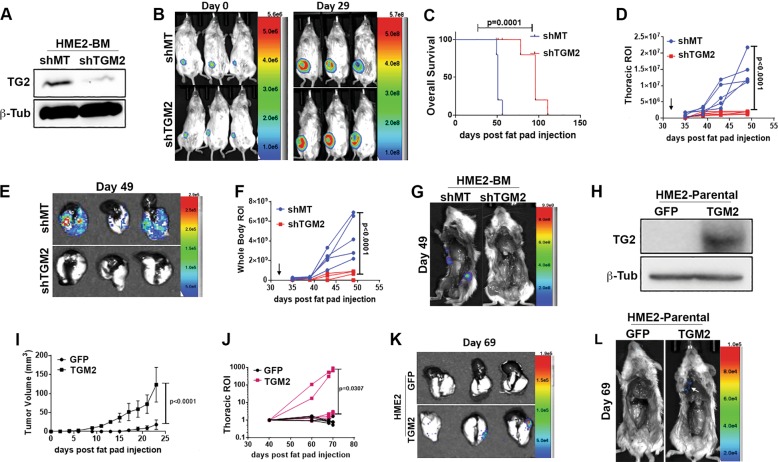
Transglutaminase-2 drives metastasis. **a** Immunoblot analyses for TG2 in HME2-BM cells expressing TG2-targeted shRNAs (shTG2) or an empty vector (shMT) as a control. Expression of β-tubulin (β-Tub) served as a loading control. **b** Cells described in **a** were engrafted onto the mammary fat pad of two separate groups of mice. Bioluminescent images were taken immediately after engraftment (Day 0) and 29 days later (Day 29). **c** Comparison of overall survival between control (shMT) and TG2-depleted (shTG2) HME-BM tumor-bearing mice. **d**–**g** Primary mammary tumors were removed 32 days after engraftment (arrows in **d** and **f**), and mice were sacrificed on day 49. Bioluminescent intensity measurements of thoracic regions of interest (ROIs; **d**) and whole-body ROI (**f**) of control (shMT) and TG2-depleted (shTG2) HME2-BM tumor-bearing mice. Upon necropsy, lungs were removed and imaged (**e**) separate of the body (**g**) to visualize pulmonary and extrapulmonary metastases. **h** Immunoblot analyses of TG2 overexpression in HME2 cells. Expression of GFP was used as a control, and β-tubulin (β-Tub) was assessed as a loading control. **i** Primary tumor growth of control (GFP) and TG2-overexpressing (TG2) HME2 tumor-bearing mice was quantified by caliper measurements. **j** Bioluminescent intensity measurements of thoracic ROIs of control (GFP) and TG2-overexpressing (TG2) HME2 tumor-bearing mice. (**k**, **l**) Upon necropsy, the lungs (**k**) and whole mouse (**l**) were imaged separately to visualize pulmonary and extrapulmonary metastases. Data in **c**, **d**, **f**, **i**, **j** are the mean ± SE or individual values of five mice per group resulting in the indicated *p* values. Data in **j** are the bioluminescent radiance values for each mouse.

To determine whether TG2 is functionally involved in metastasis, we depleted its expression in the HME2-BM cells and engrafted these cells onto the mammary fat pad of NRG mice (Fig. 3a, b). Depletion of TG2 had a minimal effect on primary tumor growth but inhibited pulmonary metastasis and promoted overall and metastasis-free survival (Fig. 3c–g, Supplementary Fig. 1a). To examine the sufficiency of TG2 in promoting disease progression, we overexpressed it in the parental HME2 cells and similarly assessed in vivo tumor growth and metastasis (Fig. 3h). In contrast to depletion of TG2 in the HME2-BM cells, overexpression of TG2 in HME2 cells did significantly increase the growth rate of primary tumors (Fig. 3i, Supplementary Fig. 1b, c). More importantly, we were able to observe pulmonary metastasis in TG2-overexpressing HME2 cells, a result we have yet to observe from parental HME2 tumors in this and other studies (Fig. 3i–l, Supplementary Fig. 1b–d)^3,20^.

**Fig. 4:**
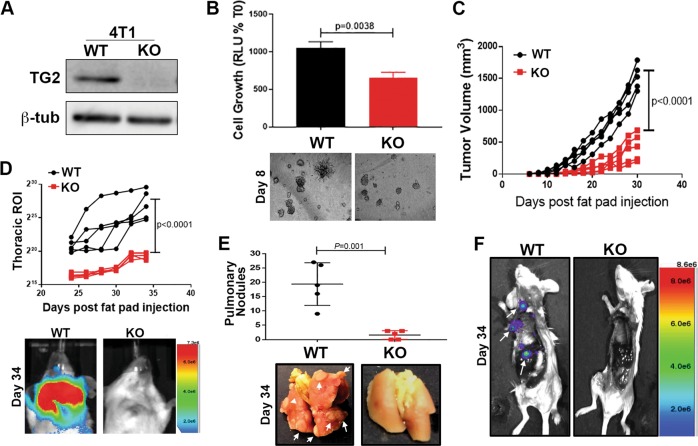
Deletion of Transglutaminase-2 inhibits metastasis. **a** Immunoblot for TG2 in control, wild-type (WT), and *TGM2*-deleted (KO) 4T1 cells. Expression of β-tubulin (β-tub) was used as a loading control. **b** Control (WT) and *TGM2*-deleted (KO) 4T1 cells were seeded under single-cell 3D culture conditions. Initiation of 3D outgrowth was quantified by bioluminescence. Data are normalized to the plated values and are the mean ± SD of three independent analyses resulting in the indicated *p* value. (below) Representative brightfield images of each 3D culture. **c** Control (WT) and *TGM2*-deleted (KO) 4T1 cells were engrafted onto the mammary fat pad and primary tumor growth was quantified by caliper measurements. Data are of individual mice taken at the indicated time points, resulting in the indicated *p* value. **d** Quantification of bioluminescent radiance from the pulmonary regions of interest (ROIs) at the indicated time points. (below) Representative thoracic bioluminescent images of control 4T1 (WT) *TG2*-deleted (KO) tumor-bearing mice. **e** Upon necropsy, the numbers of metastatic pulmonary nodules was quantified from control (WT) and *TGM2*-deleted (KO) 4T1 tumor-bearing mice. (below) Representative gross anatomical images of lungs from these groups. **f** Upon necropsy, the lungs and primary tumors of mice bearing control (WT) and *TGM2*-deleted (KO) 4T1 tumors were removed, and the carcasses were immediately imaged to visualize extra pulmonary metastases (arrows). A representative mouse from each group is shown. For **d**, **e**, data are the mean ± SE of five mice resulting in the indicated *p* values.

To validate these observations in an additional model of breast cancer metastasis, we deleted TGM2 using a CRISPR-mediated gene editing approach in the highly metastatic 4T1 cells (Fig. 4a). We have previously established that 4T1 cells efficiently interconvert between epithelial and mesenchymal phenotypes during tumor growth and metastasis^3,21^. Consistent with these studies and our previous results herein, the 4T1 cells expressed readily detectable levels of TG2 and its deletion hindered 4T1 outgrowth under single-cell 3D culture conditions (Fig. 4b and Supplementary Fig. 2a). Deletion of TG2 also inhibited primary tumor growth and the pulmonary and extrapulmonary metastasis of the 4T1 cells (Fig. 4c–f and Supplementary Fig. 2b). These results are consistent with the notion that TG2 is both necessary and sufficient to promote breast cancer metastasis^22^.

### Transglutaminase-2 crosslinks FN on EVs

**Fig. 5:**
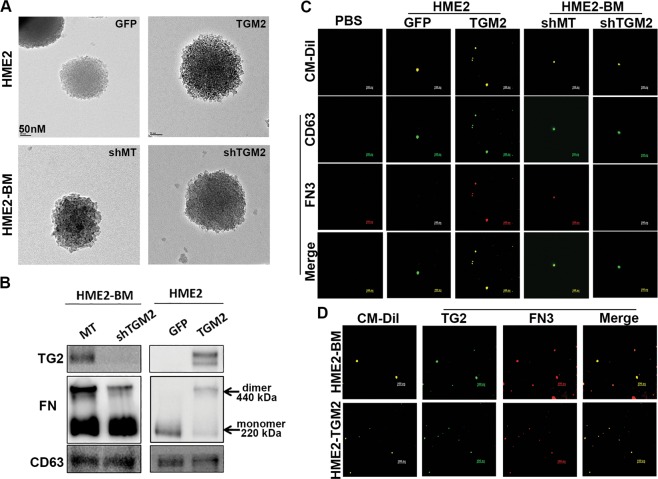
Transglutaminase-2 promotes fibronectin crosslinking on the surface of extracellular vesicles. **a** Transmission electron micrographs of extracellular vesicles derived from control (GFP) and TG2-overexpressing (TGM2) HME2 cells as well as control (shMT) and TG2-depleted (shTGM2) HME2-BM cells. **b** Immunoblot analysis of EVs derived from the HME2 and HME2-BM cells described in **a**. Differential expression of TG2 was verified in these EV lysates and correlated with covalent linkage of FN dimers that are insensitive to reducing conditions of the SDS-PAGE. CD63 served as a loading control. **c** Extracellular vesicle preparations derived from the cell types described in **a** were stained with CM-Dil (yellow) to verify the presence of lipid-containing particles. These preparations were also stained with antibodies specific for CD63 (green) and FN3 (red) and imaged using confocal microscope. The green (CD63) and red (FN3) channels were merged. A blank control sample (PBS) stained with the CM-Dil and appropriate secondary antibodies is also shown. **d** Extracellular vesicles derived from HME2-BM and HME2-TGM2 cells were stained with CM-Dil (yellow) and antibodies specific for TG2 (green) and fibrillar FN (FN3; red) and imaged using a confocal microscope. The green (TG2) and red (FN3) channels were merged. Scale bars on **c**, **d** are 500 nm.

Previous studies indicate that TG2 and FN are present in EVs derived from cancer cells^15,23^. We therefore isolated EVs using a 200-nM filter cut-off from non-metastatic and metastatic breast cancer cells and conducted nanoparticle tracking analysis and transmission electron microscopy (TEM) to validate vesicle isolation (Fig. 5a and Supplementary Fig. 3a). These EV fractions were further analyzed by immunoblot for the expression of TG2 and the crosslinked status of FN (Fig. 5b). Crosslinked FN dimers were only observed in vesicles derived from the HME2-BM cells that had undergone EMP, not in vesicles from the HME2 parental cells (Fig. 5b). Genetic depletion of TG2 or pharmacological inhibition of its activity using the small molecule NC9 inhibited FN crosslinking on EVs (Fig. 5b, Supplementary Fig. 3b). Finally, overexpression of TG2 was sufficient to induce FN dimerization on EVs and this could be readily inhibited by NC9 (Fig. 5b, Supplementary Fig. 3b). To further investigate the hypothesize that crosslinked FN progresses to a fibrillar form on the surface of EVs, we conducted high-magnification confocal microscopy on nonpermeabilized EVs using a lipophilic dye together with antibodies specific for TG2 and fibrillar FN (Fig. 5c, d). Results from this approach indicate that fibrillar FN exists on the surface of EVs in a TG2-dependent manner (Fig. 5b–d). Taken together, these data indicate that TG2-mediated crosslinking promotes FN fibrillogenesis on the surface of EVs derived from metastatic breast cancer cells that have undergone EMP.

### Tensin-1 (TNS1) is required for FN fibrillogenesis on EVs

**Fig. 6:**
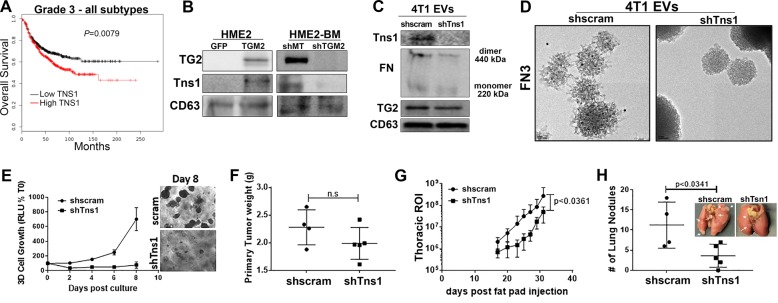
Tensin-1 is required for fibronectin fibrillogenesis on EVs. **a** Comparison of overall survival between patients bearing grade 3 tumors expressing levels of TNS1 above (high) or below (low) the mean level for the entire patient cohort. Survival curves were analyzed via a log-rank test resulting in the indicated *p* value. **b** Immunoblot analyses of EVs collected from control (GFP) and TG2-overexpressing HME2 (TGM2) as well as control (shMT) and TG2-depleted (shTGM2) HME2-BM cells. Lysates from these EVs were assessed for the levels of TG2 and TNS1, and the levels of CD63 served as a loading control. **c** Immunoblot analyses of EVs derived from control (shscram) and Tns1-depleted (shTns1) 4T1 cells. Lysates from these EVs were assessed for the levels of TG2 and Tns1, and the levels of CD63 served as a loading control. **d** The fibrillar FN-specific (FN3) antibody was used in conjunction with immuno-electron microscopy to analyze nonpermeabilized EVs derived from control (shscram) and Tns1-depleted (shTns1) 4T1 cells. **e** Control (shscram) and Tns1-depleted (shTns1) 4T1 cells were grown under single-cell 3D culture conditions. Cellular outgrowth was quantified by bioluminescence at Day 8. Data are normalized to the plated values and are the mean ± SD of three independent analyses resulting in the indicated *p* value. **f** Control (shscram) and Tns1-depleted (shTns1) 4T1 cells were engrafted onto the mammary fat pad, and the resultant primary tumors were removed and weighed upon necropsy. **g** Quantification of bioluminescent radiance from the thoracic regions of interest (ROIs) at the indicated time points. **h** Upon necropsy, the numbers of pulmonary metastatic nodules were quantified. (Inset) Corresponding gross anatomical views of lungs from control (shscram) and Tns1-depleted (shTns1) 4T1 tumor-bearing mice. Data in **f**–**h** are mean ± SE values from five mice per group resulting in no significance (n.s.) or the indicated *p* values.

Our recent phospho-mass spectrometric analyses indicate increased phosphorylation of TNS1 in EVs derived from the serum of breast cancer patients as compared to healthy individuals^24^. Furthermore, Kaplan–Meier (KM) analyses indicate that enhanced expression levels of TNS1 are strongly associated with decreased survival of advanced-stage breast cancer patients (Fig. 6a). Mechanistically, TNS1 plays an important role in FN fibrillogenesis by binding to and promoting clustering of ECM-bound integrins^25^. Consistent with these data, TNS1 was readily detectable in HME2 EV fractions and was markedly dependent on the expression of TG2 (Fig. 6a, Supplementary Fig. 4a). In contrast, targeted depletion of Tns1 in the 4T1 cells did not affect TG2 presence on EVs or FN dimerization (Fig. 6c, Supplementary Fig. 4b). However, use of the fibrillar FN-specific antibody in conjunction with immuno-electron microscopy indicated that FN fibrillogenesis was not achieved on EVs in the absence of Tns1 (Fig. 6d). These data suggest that TG2 can crosslink FN on EVs but completion of FN fibrillogenesis requires the presence of TNS1. Functionally, depletion of Tns1 inhibited the outgrowth of the 4T1 cells under single-cell 3D culture conditions (Fig. 6e). Upon orthotopic engraftment, Tns1 depletion had no effect on primary tumor growth but did significantly inhibit pulmonary metastasis (Fig. 6f–h). Together with findings from the previous figures, these data suggest that TG2 mediates the presence of TNS1 in tumor-derived EVs, leading to FN fibrillogenesis and promotion of metastasis.

### Transglutaminase-2 promotes EV-mediated metastatic niche formation

**Fig. 7:**
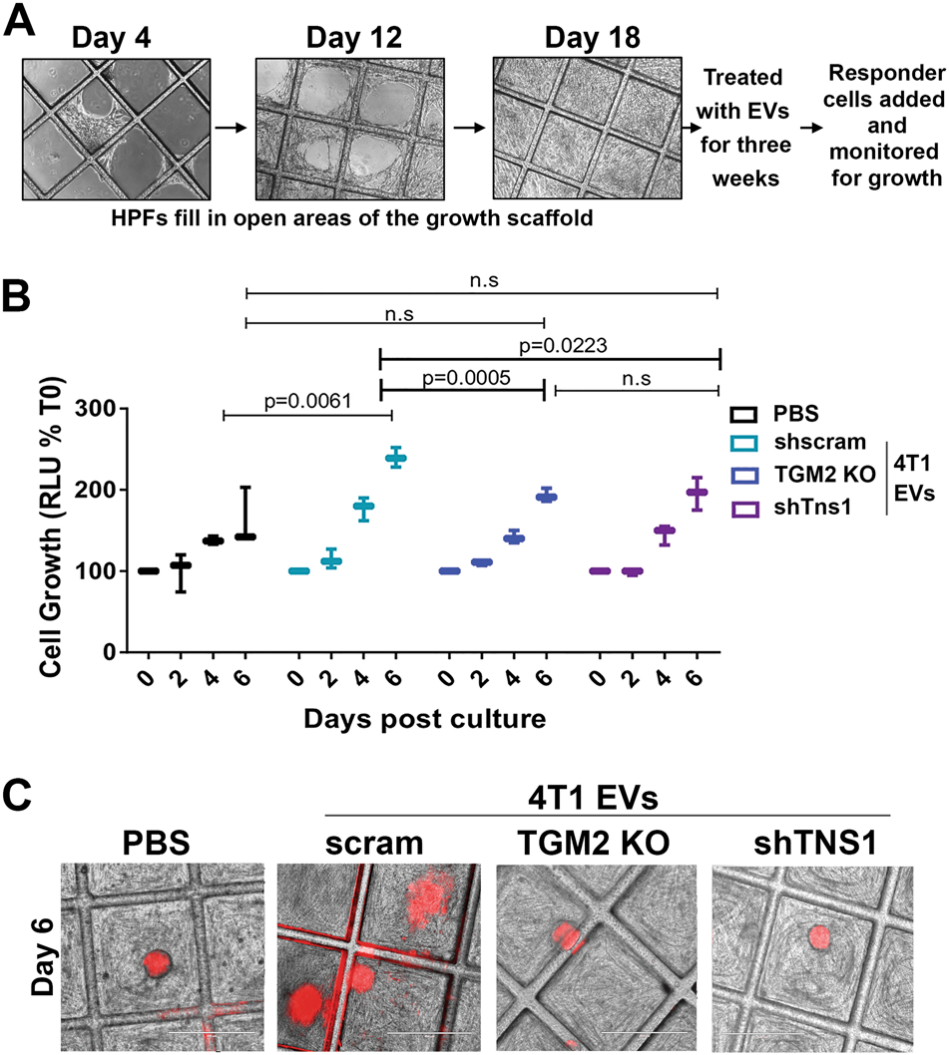
Transglutaminase-2 promotes EV-mediated pulmonary niche formation. **a** Schematic illustration of the 3D niche assay. Human pulmonary fibroblasts (HPFs) were cultured on 3D scaffolds as described in the “Materials and methods” section and allowed to fill the open space of tessellated polymeric scaffolds. These cultures were treated with exosomes for another 3 weeks at which point MCF10-Ca1a responder cells stably expressing a firefly luciferase-dTomato fusion protein were added to the culture. **b** Growth of labeled Ca1a cells was longitudinally quantified by bioluminescence at the indicated time points. Data are mean relative luminescence units (RLU), normalized to the time zero (T0) reading, ±SE for three independent experiments completed in triplicate resulting in the indicated *p* values. **c** Representative fluorescent and brightfield merged images showing MCF10-Cala cell colonies (red) growing on the HPFs.

To focus on the specific role of EVs in TG2 and TNS1-mediated metastasis, we developed a coculture model of the pulmonary niche. To do this, human pulmonary fibroblasts (HPFs) were grown to confluence on our recently described tessellated polymeric scaffolds to create a 3D platform for subsequent coculture with tumor cells (Fig. 7a)^9^. The HPFs were treated with EVs derived from control, TG2-deleted, or TNS1-depleted 4T1 cells for 3 weeks in attempts to modulate the growth environment (Fig. 7a). Following pretreatment with these EVs, responder cells (MCF10-Ca1a cells expressing a firefly luciferase-dTomato fusion protein) were seeded onto the HPFs (Fig. 7a). Using this approach, we observed that HPFs pretreated with wild-type (WT) 4T1-derived EVs significantly enhanced the growth of responder cells as compared to untreated HPFs (Fig. 7b, c). Moreover, depletion of TG2 or TNS1 significantly reduced the ability of EVs to induce the growth-supportive phenotype of the HPFs (Fig. 7b, c).

**Fig. 8:**
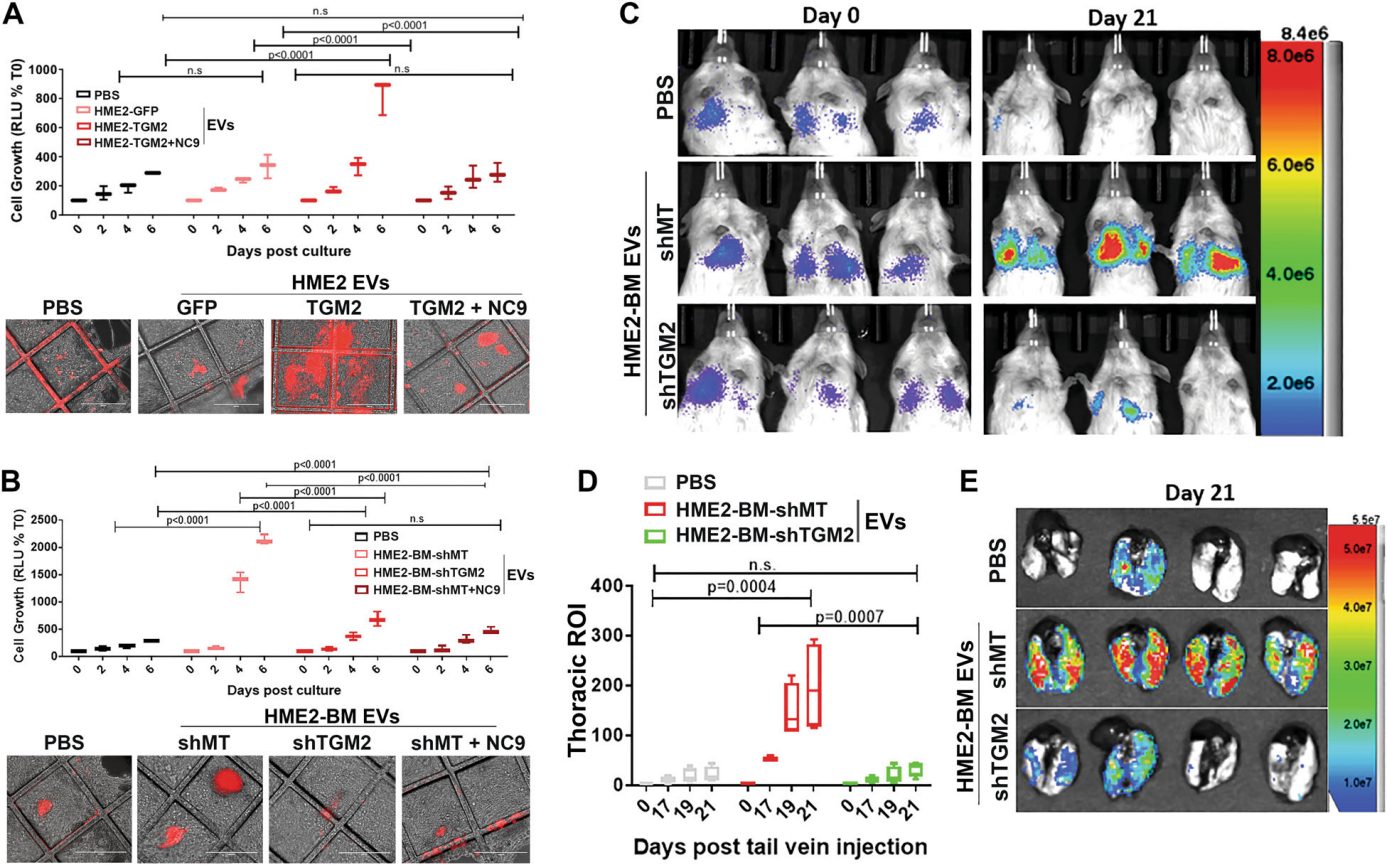
Transglutaminase-2 enhances the ability of EVs to increase pulmonary colonization. **a**, **b** Three-dimensional cultures of human pulmonary fibroblasts were treated with the indicated EVs as described in the “Materials and methods” section. MCF10-Ca1a cells stably expressing a firefly luciferase-dTomato fusion protein were subsequently added to these cultures, and their growth was longitudinally quantified by bioluminescence at the indicated time points. Data are mean relative luminescence units (RLU), normalized to the time zero (T0) reading, ±SE for three independent experiments completed in triplicate resulting in the indicated *p* values. Representative fluorescent and brightfield merged images showing Cala cell colonies (red) growing on the HPFs. **c** Mice were pretreated with the indicated EVs via intraperitoneal injections for 3 weeks prior to tail vein administration of the labeled MCF10-Ca1a cells described in **a**. Bioluminescent images of representative mice taken immediately (Day 0) and 21 days (Day 21) following tail vein injection of the MCF10-Ca1a cells. **d** The mean (±SE) bioluminescence values of thoracic regions of interest (ROIs) taken at the indicated time points. Data are normalized to the injected values, *n* = 4 mice in each group resulting in the indicated *p* value. **e** Mice were sacrificed at day 21, and upon necropsy, lungs were imaged ex vivo using bioluminescence to visualize pulmonary tumor formation.

We next utilized this 3D coculture system to examine the ability of EVs from HME2 cells to promote the growth of responder cells. Indeed, HPFs pretreated with EVs derived from parental HME2 cells failed to increase the subsequent growth of responder cells, but this could be drastically enhanced by overexpression of TG2 (Fig. 8a). Conversely, pretreatment of HPFs with HME2-BM-derived EVs promoted responder cell growth, and this was prevented upon depletion of TG2 in the EVs (Fig. 8b). Importantly, prevention of FN crosslinking on EVs via treatment of EV-producing cells with NC9 also prevented the ability of the resultant EVs to induce the growth-supportive phenotype of the HPFs (Fig. 8a, b). To confirm that EVs derived from metastatic breast cancer cells promote metastatic niche formation in a TG2-dependent manner, NSG mice were pretreated with WT and TG2-depleted EVs via intraperitoneal injections for 3 weeks. These mice were then subsequently given tail vein injections of the bioluminescent MCF10-Ca1a responder cells. Consistent with our 3D coculture results, in vivo administration of HME2-BM-derived EVs enhanced the pulmonary colonization of the responder cells (Fig. 8c–e). Importantly, this effect was abolished when TG2 was depleted from the administered EVs (Fig. 8c–e). Together, these results suggest that EVs derived from metastatic breast cancer cells utilize the aberrant presence of TG2 to educate pulmonary fibroblasts to form a pulmonary niche more suitable for metastatic colonization.

## Discussion

Several recent studies from our laboratory and others indicate that dynamic induction and reversion of EMP drives tumor cell heterogeneity and supports more efficient completion of several steps of the metastatic process^8,9,26^. Herein we present a global characterization of gene expression changes that only emerge after cells complete EMP. This approach led to the discovery of TG2 upregulation as a marker of EMP. We went on to validate the necessity and sufficiency of TG2 in promoting metastasis and explored the impact of TG2 on FN fibrilization and function of EVs in producing a metastatic niche.

TG2 has previously been linked to EMT, and in contrast to our observations, its expression has been observed in cells with a mesenchymal morphology^27^. These data clearly suggest model-dependent changes in gene expression that occur during EMP. For instance, TGF-β stimulation of the HME2 model results in a mesenchymal phenotype, which, upon cessation of cytokine treatment, is fully capable of transitioning back to an epithelial morphology indistinguishable from the original cells^28^. In contrast, different cell models or differences in EMT-inducing stimuli may result in EMT events that have a spectrum of gene expression and morphological reversion capabilities. Indeed, using the HME2 model we recently established that chronic inhibition of HER2 kinase activity with the drug lapatinib results in an EMT that does not revert upon removal of the drug^28^. Therefore, some EMT events may revert to an extent that includes upregulation of TG2, or other genes, but may not include an overt morphologic change back to an epithelial state. This concept would serve to explain why certain cell models that have a stable mesenchymal morphology are fully capable of completing metastasis, while others fail to complete the latter stages of metastatic outgrowth. Overall, our findings in the HME2 model highlight the importance of defining EMT and MET by quantitative changes in gene expression or other metrics, not by morphologies that manifest under two-dimensional culture conditions.

Additional complexity in regard to the role of TG2 in EMT may also stem from its diversity in function both inside and outside the cell^29^. Using the HME2 and 4T1 model systems, we demonstrate that depletion of TG2 consistently inhibits metastasis but can have variable impacts on primary tumor growth. Therefore, we focused on characterizing the systemic influence of TG2-containing EVs in the development of metastatic niches with increased capacity to support systemic tumor cell seeding and outgrowth^30^. Both FN and TG2 have been observed in EVs derived from breast cancer cells^15,31^. In this study, we demonstrate the necessity and sufficiency of TG2 to generate fibrillar FN on the surface of EVs, an event that fosters pulmonary niche formation and supports the subsequent colonization of breast cancer cells. These functional data are supported by our previous patient analyses that utilized mass spectrometry to identify increased phosphorylation of TNS1 in EVs derived from breast cancer patients as compared to healthy controls^24^.

EVs derived from cancer cells contain multiple biologically active molecules including proteins, nucleic acids, and lipids that can alter local stroma to create a disease-supportive microenvironment. Our studies utilize a novel, tessellated 3D coculture platform to establish that TG2 is required for EV-mediated modification of pulmonary fibroblasts. However, important questions remain with regard to the precise mechanism by which TG2 and fibrillar FN participate in this process. Comprehensive analyses are required to characterize changes in the EV components upon TG2 depletion and/or inhibition in cells that have undergone EMP^15^. Moreover, the presence of fibrillar FN on the surface of EVs could drastically alter the amount and route of EV internalization into pulmonary fibroblasts, leading to pathologic alteration of fibroblast gene expression and function. Finally, delivery of TG2 and FN into the stromal pulmonary microenvironment may be sufficient to alter the existing matrix into a more tumor-permissive state. Studies in our laboratory are currently ongoing to better delineate such mechanisms by which the presence of TG2 alters EV function.

Clinically, our data suggest that attempts to quantify systemic changes in TG2 activity as a bodily fluid biomarker could be augmented by the preparation of EV fractions prior to these analyses^32,33^. Finally, our studies also support the notion that targeted inhibition of TG2 will limit disease progression by not only preventing local fibrotic reactions but also through inhibition of metastatic niche formation. Overall, our data present a comprehensive characterization of EMP and illustrate a novel impact of this process on EV function and metastatic progression.

## Materials and methods

### Reagents

The murine metastatic 4T1 cells were constructed to stably express firefly luciferase via stable transfection under Zeocin selection^34^. The HMLE cells were constructed to stably express firefly luciferase via viral transduction under Blasticidin selection; these cells were subsequently transformed by overexpression of HER2 via stable transduction under puromycin selection, yielding the parental HME2 cell line. The HME2 mesenchymal variants were established via 4 weeks of continuous stimulation with TGF-β1 (5 ng/ml). Following a 2-week period of recovery from TGF-β stimulation, these HME2 cells were engrafted onto the mammary fat pad of immunocompromised mice and subsequent bone metastases were subcultured, yielding the HME2-BM cell line^3,20^. The MCF10-Ca1a cells were constructed to stably express a fusion protein of d-Tomato Red and firefly luciferase via stable transduction under Zeocin selection^9^. Manipulation of TG2 expression was achieved through lentiviral-mediated transduction of TRCN0000000239, TRCN0000000241, or a scrambled control short hairpin RNA, empty plko.1 vector (GE Dharmacon, Lafayette, CO). The dimeric CRISPR RNA-guided Fokl nuclease and Csy4-based multiplex gRNA expression system as previously described was used to generate the TG2 knockout 4T1 cell line^3^. Full-length human TG2 or green fluorescent protein as a control were expressed via a lentiviral delivery of pLV (Vector Builder, Santa Clara, CA). Manipulation of Tns1 expression was achieved through lentiviral-mediated transduction of V2LMM_4992 or a scrambled control (vector builder). Stable genomic integration of constructs was selected for using puromycin or hygromycin. Primary HPFs were obtained from ATCC and cultured in the recommended fibroblast basal media supplemented with fibroblast growth factor low serum kit (ATCC). All cell lines were authenticated and tested for mycoplasma contaminated via the IDEXX IMPACT III CellCheck in December of 2018. The TG2 small molecule inhibitor (nc-9) was a kind gift from Dr. Jefferey Keillor (University of Ottawa).

### Gene expression analysis

Gene expression data for the HME2 parental, TGF-β1-treated, and HME2-BM cells were extracted from GSE115255 as previously described^3^. Afterwards, the raw counts were imported to the R environment of statistical computing (v3.6.0). Differentially expressed genes were determined by using edgeR (v3.26.4, refs. ^35,36^) and defined as having a log fold change >2 and adjusted *p* value <0.05. Then the genes were grouped using unsupervised clustering via *k*-means, determining the optimal *k* by the elbow method and plotted using the R packages Complex Heatmap (v2.0.0, ref. ^37^) or ggplot2 (10.1007/978-0-387-98141-3). For RT-PCR, total RNA was reverse-transcribed using a cDNA synthesis kit (Thermo Fisher). Semi-quantitative real-time PCR was performed using iQ SYBR Green (Thermo Fisher). The following primers were used for the analysis. *TGM2* sense; ATAAGTTAGCGCCGCTCTCC, *TGM2* antisense; CTCTAAGACCAGCTCCTCGG; *Tns1* sense; CACCGTGAGCCTGGTGTG, *Tns1* antisense; AGGCCTTCACCTTGAAGT.

### Animal models

All in vivo assays were conducted under IACUC approval from Purdue University. Where indicated, luciferase expressing HME2 variants (2 × 10^6^/50 µl) were injected into the second mammary fat pad of female 8-week-old, NSG mice. Tumors were surgically excised after they achieved the size of 900 mm, and metastasis was subsequently quantified by bioluminescent imaging using the Advanced Molecular Imager (AMI) (Spectral Instruments, Tucson, AZ). For EV preconditioning experiments, 20 µg of total EV protein in 100 µl of sterile phosphate-buffered saline (PBS) was injected intraperitoneally into 3-month-old NSG mice every other day for 3 weeks. Mice received 100 µl of sterile PBS in the control group. Luciferase expressing Ca1a (7.5 × 10^5^/100 µl) were injected into the lateral tail vein of EV-pretreated female NSG mice. Pulmonary tumor growth was quantified by bioluminescence at the indicated time points.

Luciferase expressing 4T1 cells were resuspended in PBS (50 µl) and orthotopically engrafted onto the second mammary fat pad of randomized 4-week-old Balb/c mice (2.5 × 10^4^ cells/mouse) (Jackson Laboratories, Bar Harbor, ME). Primary tumor growth and metastasis development were assessed via weekly bioluminescent imaging. Upon necropsy, lungs from all animals were removed and fixed in 10% formalin and dehydrated in 70% ethanol for visualization of pulmonary metastatic nodules and histological analyses. Investigators were not blinded to the conditions of the study.

### Isolation of EVs

EVs were isolated from 72-h, serum-free conditioned media of 10^7^ cells (equivalent to four 150-mm dishes) as described previously^38,39^. Briefly, conditioned media was centrifuged at 300 × *g* for 10 min to remove live cells and then 2000 × *g* for 10 min to remove cell debris. This supernatant was filtered through a 0.22-µM pore size Millipore filter. Filtered media was concentrated to 1 ml using 3-KDa molecular weight cut-off (MWCO) Amicon ultra-15 centrifugal filters, followed by ultracentrifugation at 100,000 × *g* for 2 h. The pellet was washed with PBS using ultracentrifugation at 100,000 × *g* for an additional 2 h. The pelleted EVs were resuspended either in 3D RIPA buffer for immunoblot experiments or in PBS for biological or imaging experiments. Size distribution and concentration of EVs were analyzed via semiautomated nanoparticle tracking using a NanoSight (NS300) apparatus (Malvern Instruments Ltd.). Samples were diluted to provide counts within the linear range of the instrument (3 × 10^8^–1 × 10^9^/ml). Three videos of 1-min duration were documented for each sample, with a frame rate of 30 frames/s. Using the NTA software (NTA 2.3; NanoSight Ltd.), particle movement was analyzed as per the manufacturer’s protocol. The NTA software was adjusted to first identify and then track each particle on a frame-by-frame basis.

### Immunological assays

Protein expression on the surface of EVs was examined using whole-mount immunostaining as described previously^40^. Briefly, thin formvar/carbon film-coated 200 mesh copper EM grid were glow discharged for 30 s. EVs were fixed in 1 ml of paraformaldehyde for 5 min. In all, 5–7 µl of fixed EVs were loaded onto the grids and incubated for 10 min. The grids were rinsed with 100 µl of PBS three times each for 10 min, then treated with 50 µl of glycine to quench free aldehyde groups for 10 min. The grid was then incubated with 100 µl of blocking buffer (PBS containing 1% bovine serum albumin (BSA)) for 30 min and finally incubated with 100 µl of primary antibody (anti-FN3 (1:100)) overnight at 4 °C. The following day, the grids were washed with 100 µl of washing buffer (PBS containing 0.1% BSA) five times each for 10 min. Grids were incubated with secondary antibodies conjugated to 10-nm gold particle (ab39619, abcam) diluted at 1:100 in PBS containing 0.1% BSA for 1 h. Grids were washed with washing buffer five times each for 10 min and 50 µl of distilled water twice. Grids were air dried for 15 min and observed via TEM at 200 kV.

For confocal microscopic imaging, EV samples were prepared as described previously^9^. Briefly, 500 µl of PBS solution containing EVs was incubated simultaneously with 2 µl each of antibodies specific for CD63 (ab217345), TG2 (Invitrogen CUB 7402), and FN3 (Invitrogen 14-9869-82) for 2 h at room temperature. Following incubation, the solution was purified by ultrafiltration (50 KDa MWCO) at 600 rpm for 20 min. The filtrate was washed with PBS using ultrafiltration and resuspended in PBS. Next, a mixture of 0.5 µl of CM-dil, 1 µl of 2 mg/ml of Alexa Fluor-labeled 647 Goat anti-mouse IgG, and 1 µl of 2 mg/ml of Alexa Fluor 488-labeled Goat anti-rabbit IgG was added to the EV solution, incubated for 1 h with vigorous mixing, and then purified again by ultrafiltration. Finally, the precipitate was resuspended in PBS and added to 35 mm^2^ 1.5H glass coverslip bottom confocal dish and adsorbed for 15 min. The dishes were coated with 0.1% polyethylenimine for 15 min prior to addition of the prepared sample. Samples were imaged using Nikon confocal microscope.

For immunoblot analyses, cells and EV fractions were lysed using a modified RIPA lysis buffer containing 50 mM Tris, 150 mM NaCl, 0.25% sodium deoxycholate, 1.0% NP40, 0.1% sodium dodecyl sulfate (SDS), protease inhibitor cocktail, 10 mM activated sodium ortho-vanadate, 40 mM β-glycerolphosphate, and 20 mM sodium fluoride. These lysates were separated by reducing SDS polyacrylamide gel electrophoresis and probed for TG2 (Invitrogen; CUB7402), TNS1 (Sigma; HPA036089), CD63 (Santa Cruz; sc-5275), FN (BD biosciences; 610078), Ecad (BD biosciences, 610182), FN3 (eBiosciences; 14-9868-82), or β-tubulin (DSHB, E7-s).

### 3D scaffold assays

Scaffolds were constructed as described previously^9^. Uncoated 3D scaffolds were placed in ultralow attachment 24-well dishes. HPFs (100,000) were added to the scaffolds. Cells were fed new media every 5 days for 2–3 weeks. Once the scaffolds were entirely covered with HPFs, they were treated with 5 µg of EVs every other day for 3 weeks. Ca1a FF-dTomato cells (50,000) were added to the EV-pretreated scaffolds and tracked for growth using bioluminescence and fluorescent imaging at the indicated time points.

### Statistical analyses

KM analyses of patient survival were conducted using the KM-plotter platform (http://www.kmplot.com) where *p* values are representative of a log-rank analysis. Two-way analysis of variance or two-sided *T* tests were used where the data met the assumptions of these tests and the variance was similar between the two groups being compared. For all studies, sample sizes were chosen based on a power analysis given previously established variance for each experimental approach. *p* Values of < 0.05 were considered significant. No exclusion criteria were utilized in these studies.

## Supplementary information

Supplemental Figure Legends

Supplemental Figure 1

Supplemental Figure 2

Supplemental Figure 3

Supplemental Figure 4
