# Supplementary material for: Transglutaminase-2 facilitates extracellular vesicle-mediated establishment of the metastatic niche
Source: Oncogenesis. 2020 Feb 13;9(2):16. doi: 10.1038/s41389-020-0204-5 (PMC7018754; doi:10.1038/s41389-020-0204-5)
Supplement: Supplementary file 1 — Supplemental Figure Legends [file 41389_2020_204_MOESM1_ESM.docx]

**Supplementary Figure Legends:**

**Supplementary figure 1: TG2 expression promotes metastasis and decreases survival.** (A) Metastasis free survival analysis of mice orthotopically engrafted with HME2-BM MT and HME2-BM shTG2. Dotted lined indicates time point at which the primary tumor was surgically removed. (B-C) Control (GFP) and TG2 overexpressing HME2 cells were engrafted onto the mammary fat pad via an intraductal inoculation and primary tumor growth was visualized and measured by bioluminescent at the indicated time points. Data are of individual mice resulting in the indicated P value. (D) Upon necropsy the lungs of tumor bearing mice were removed and weighed. Data in panels C and D are of individual mice resulting in the indicated P value.

**Supplementary figure 2: Deletion of TG2 inhibits 3D growth and metastasis.** (A) Control (WT) and TG2 deleted (TG2KO) 4T1 cells were grown under single cell 3D culture conditions. Longitudinal cellular outgrowth was quantified by bioluminescence at the indicated time points. Data are normalized to the plated values and are the mean ±SD of three independent analyses resulting in the indicated P-value. (B) Control (WT) and TG2 deleted (TG2KO) 4T1 cells were engrafted onto the mammary fat pad and lungs were removed and weighed after mice were sacrificed at Day 34. Data are of individual mice resulting in the indicated mean, +SE and P value.

**Supplementary figure 3: TG2 crosslinks FN in tumor cell-derived EVs**. (A) Nanoparticle tracking analysis of EVs derived from the indicated cells. (B) Immunoblot analysis of EVs derived from HME2-BM MT, HME2-BM MT treated with NC9, HME2-TG2, and HME2-TG2 treated with NC9. These lysates were assessed for the presence of TG2 and FN dimerization. CD63 served as a loading control.

**Supplementary figure 4: TG2 is required for the presence of Tns1 on EVs.** (A) Extracellular vesicles were isolated from control (WT) and TG2 deleted (TG2KO) 4T1 cells. These EVs were analyzed for the presence of TG2, FN dimerization, and Tns1. CD63 served as a positive control. (B) RT-PCR analyses of Tns1 in control (shscram) and Tns1 depleted (shTns1) 4T1 cells. Data are the mean expression values of three independent samples normalized to GAPDH.
