# Supplementary figures and images for: Transglutaminase-2 facilitates extracellular vesicle-mediated establishment of the metastatic niche
Source: Oncogenesis. 2020 Feb 13;9(2):16. doi: 10.1038/s41389-020-0204-5 (PMC7018754; doi:10.1038/s41389-020-0204-5)

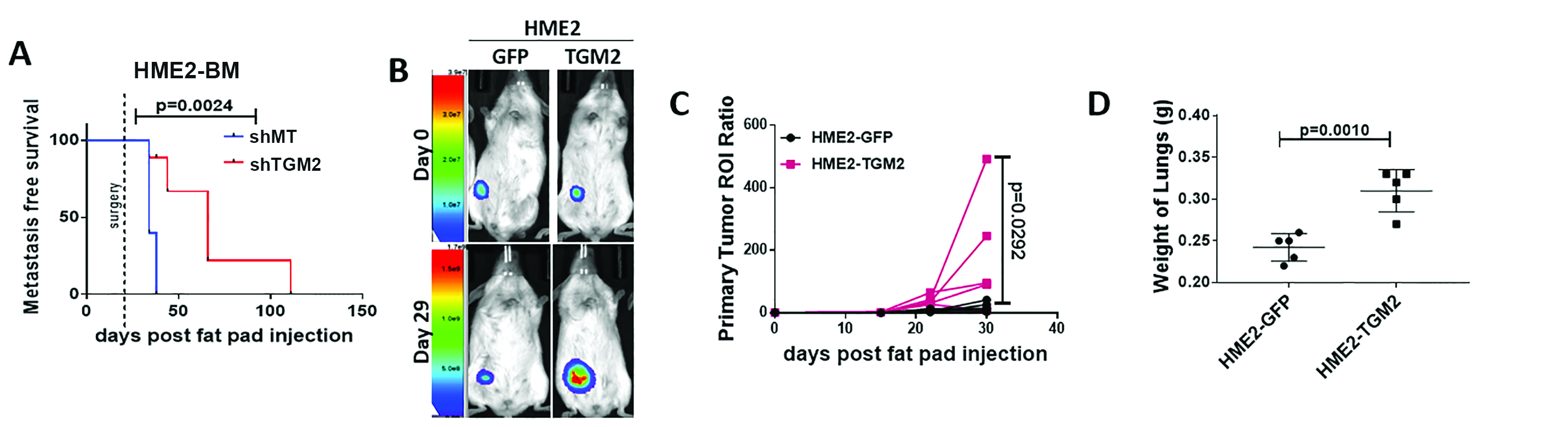

Supplement: Supplementary file 2 — Supplemental Figure 1 [file 41389_2020_204_MOESM2_ESM.tif]

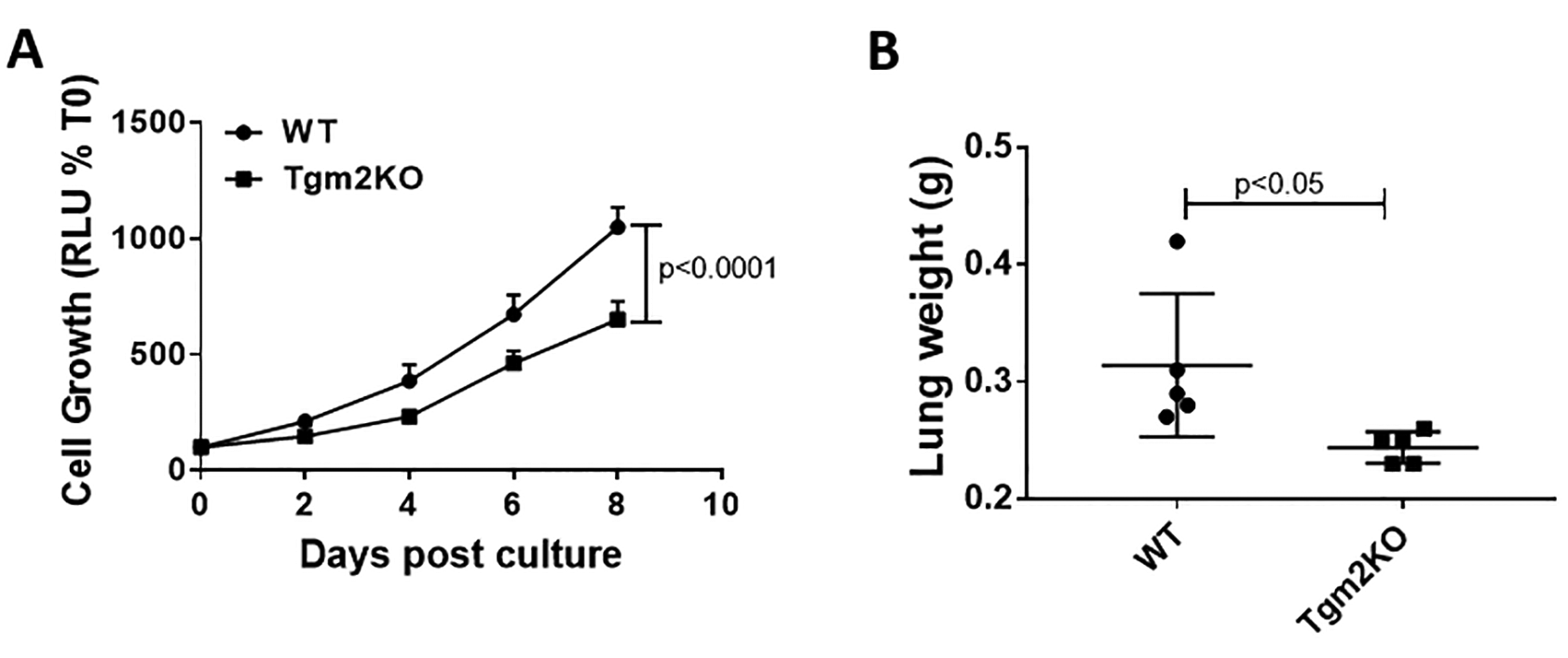

Supplement: Supplementary file 3 — Supplemental Figure 2 [file 41389_2020_204_MOESM3_ESM.tif]

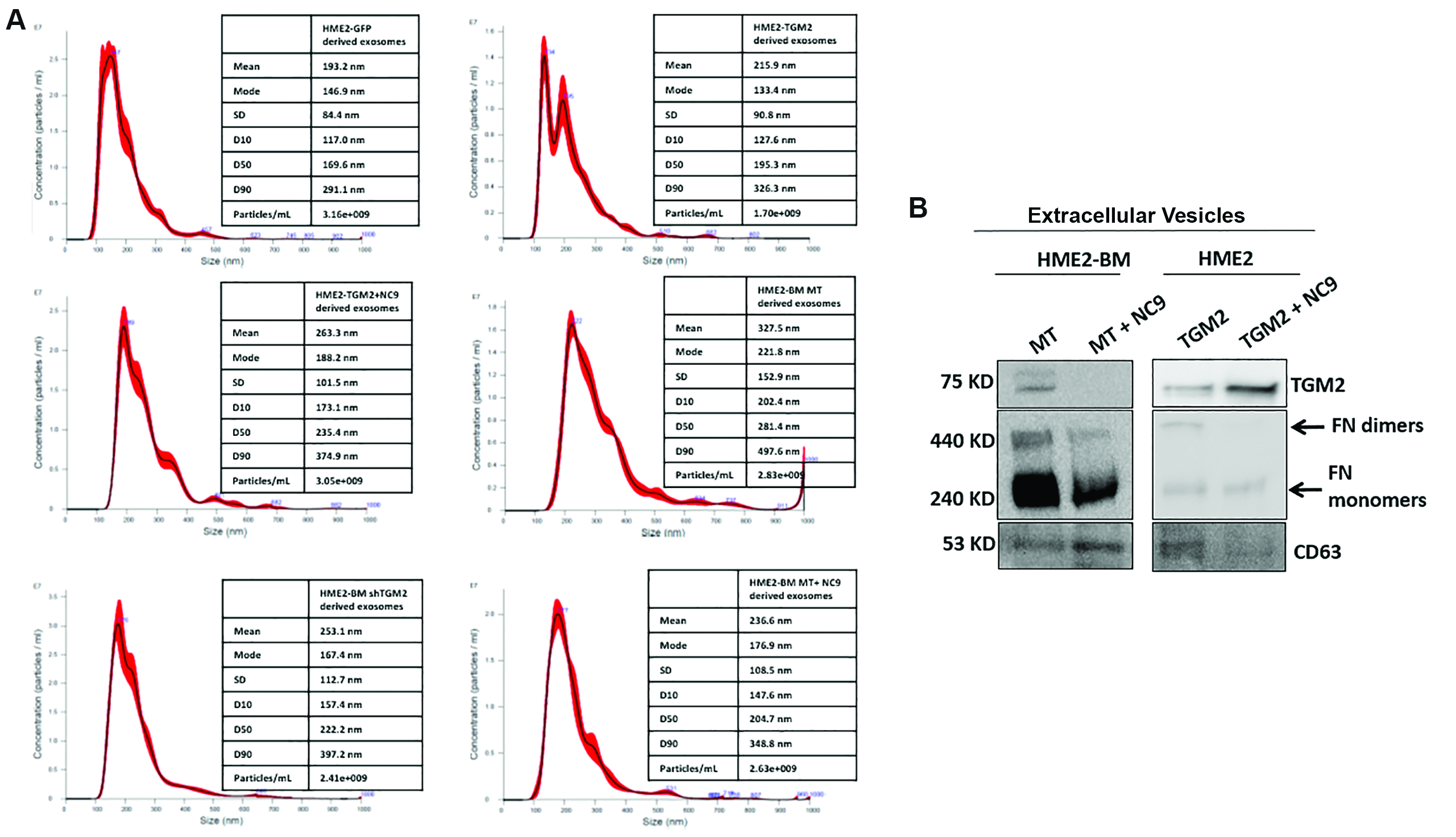

Supplement: Supplementary file 4 — Supplemental Figure 3 [file 41389_2020_204_MOESM4_ESM.tif]

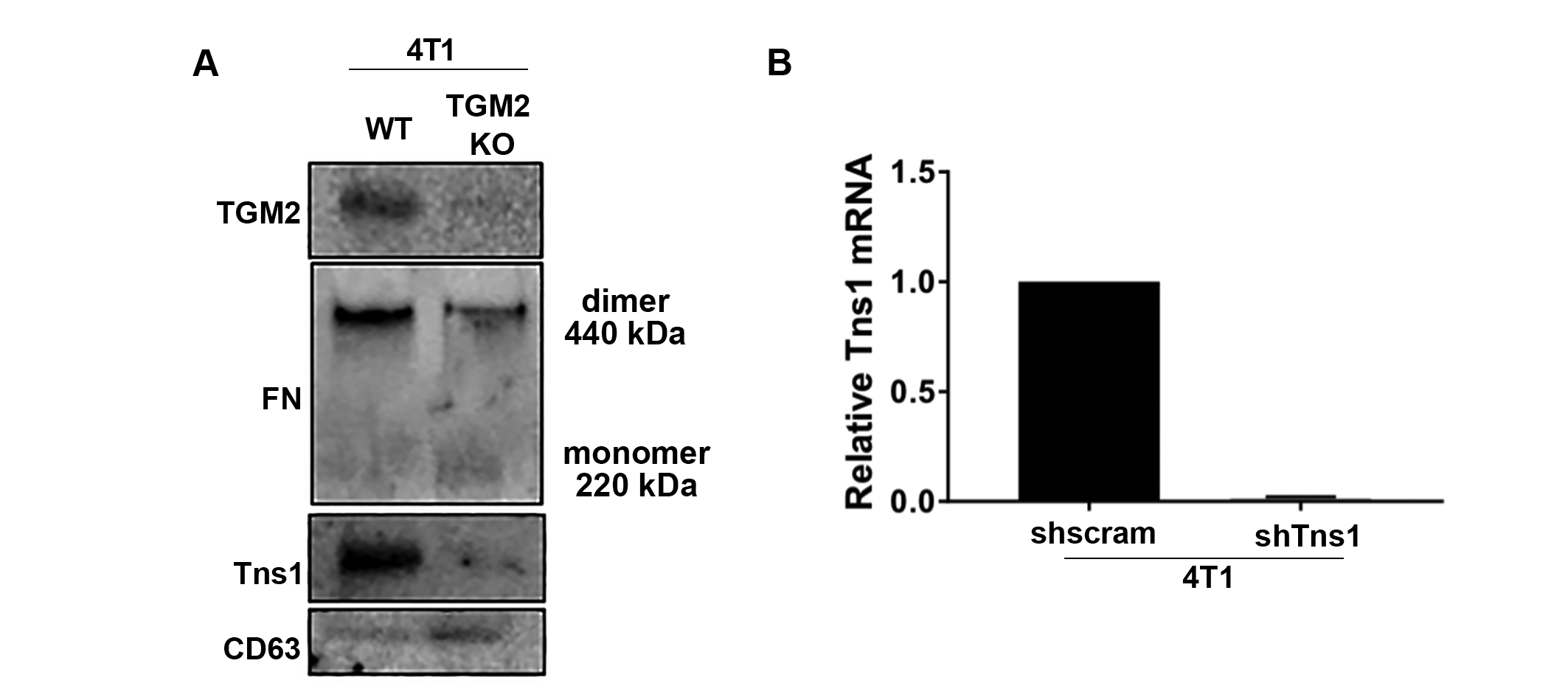

Supplement: Supplementary file 5 — Supplemental Figure 4 [file 41389_2020_204_MOESM5_ESM.tif]
